# Fluorescence-based monitoring of the pressure-induced aggregation microenvironment evolution for an AIEgen under multiple excitation channels

**DOI:** 10.1038/s41467-022-32968-9

**Published:** 2022-09-06

**Authors:** Shuang Tong, Jianhong Dai, Jiangman Sun, Yuanyuan Liu, Xiaoli Ma, Zhehong Liu, Teng Ma, Jiao Tan, Zhen Yao, Shanmin Wang, Haiyan Zheng, Kai Wang, Fang Hong, Xiaohui Yu, Chunxiao Gao, Xinggui Gu

**Affiliations:** 1 State Key Laboratory of Superhard Materials, Jilin University, Changchun, 130012 China; 2 Beijing National Laboratory for Condensed Matter Physics, Institute of Physics, Chinese Academy of Sciences, Beijing, 100190 China; 3 Beijing Advanced Innovation Center for Soft Matter Science and Engineering, College of Materials Science and Engineering, Beijing University of Chemical Technology, Beijing, 100029 China; 4 Department of Physics and Academy for Advanced Interdisciplinary Studies, Southern University of Science and Technology, Shenzhen, 518055 China; 5 Center for High Pressure Science and Technology Advanced Research, Beijing, 100094 China; 6 Songshan Lake Materials Laboratory, Dongguan, Guangdong 523808 China; 7 Beijing National Laboratory for Molecular Sciences, Beijing, 100190 China

**Keywords:** Single-molecule fluorescence, Organic LEDs, Chemical physics

## Abstract

The development of organic solid-state luminescent materials, especially those sensitive to aggregation microenvironment, is critical for their applications in devices such as pressure-sensitive elements, sensors, and photoelectric devices. However, it still faces certain challenges and a deep understanding of the corresponding internal mechanisms is required. Here, we put forward an unconventional strategy to explore the pressure-induced evolution of the aggregation microenvironment, involving changes in molecular conformation, stacking mode, and intermolecular interaction, by monitoring the emission under multiple excitation channels based on a luminogen with aggregation-induced emission characteristics of di(*p*-methoxylphenyl)dibenzofulvene. Under three excitation wavelengths, the distinct emission behaviors have been interestingly observed to reveal the pressure-induced structural evolution, well consistent with the results from ultraviolet-visible absorption, high-pressure angle-dispersive X-ray diffraction, and infrared studies, which have rarely been reported before. This finding provides important insights into the design of organic solid luminescent materials and greatly promotes the development of stimulus-responsive luminescent materials.

## Abstract (web summary)

The develop of organic functional materials requires the exploration of the pressure dependent emissive mechanisms of molecular structures, conformations and stacking modes. Here, Tong et al propose a strategy for monitoring the pressure-induced fluorescence under multiple excitation channels.

## Introduction

Solid-state luminescent materials have gained extensive attention because of their promising applications in anti-counterfeiting technology^1,2^, fluorescent probes^3–5^, sensors^6,7^, optical devices^8,9^, and biological science^10–14^. In some organic luminescent materials, planarization of the molecular conformation or aggregation results in *π*-conjugated intermolecular interactions, resulting in the formation of species that adversely affect the luminescence properties. The harmful species consume energy by opening nonradiative decay pathways, thus diminishing the luminescence^15,16^. This process, referred to as an aggregation-caused quenching effect (ACQ), severely restricts the practical application of solid-state luminescent materials^17–20^. In 2001, Tang et al. introduced a concept known as aggregation-induced emission (AIE)^21^. In certain luminescent materials with active molecular motions, nonradiative transition pathways are annihilated during molecular aggregation, resulting in significantly enhanced luminous efficiency^22,23^. Considering this phenomenon, some nonluminescent materials in the solution state were rendered emissive by inducing molecular aggregation^24–26^. The discovery of the AIE effect has helped overcome the long-term limitation of conventional luminescent materials, thereby promoting further applications of prevailing luminescent systems^27–29^. Thus, systematic studies on structure-property relationships have played an irreplaceable role in the development of solid-state luminescent materials^30,31^.

As most AIE molecules have distorted and flexible structures that do not facilitate a tight *π*–*π* packing pattern, their conformations and emission behaviors are considerably susceptible to the aggregation microenvironment, which affects electron coupling and molecular stacking^15,32–35^. Some of the early effective methods developed for regulating the aggregation microenvironment of molecules were based on heating, light irradiation, or exposure to chemical vapor^36,37^. More recently, pressurization has been employed effectively as an emerging approach for structural regulation to successfully uncover the relationship between the aggregation microenvironment and material properties^38–45^. To date, studies on luminescence behaviors in response to external pressure have been focused on two aspects: pressure-induced changes in the emission wavelength and emission intensity. On the one hand, the shifts of emission spectra accompanying color changes with good reversibility in fluorescence known as piezochromism were ascribed to the intermolecular interaction change, molecular conformation change, transformation from locally excited (LE) state to intramolecular charge-transfer (ICT) state, and intra- and inter-molecular effects caused by hydrostatic pressure, respectively^46–48^. For instance, pressure induces an increase in molecular conjugation or the transformation of *sp*^2^-hybridized domains into *sp*^3^ ones, leading to a red or blue shift of the emission^49–53^. On the other hand, any modification of the molecular arrangement, conformational flexibility, and intermolecular interactions by pressure would affect the fluorophore emission intensity in detail. For example, intermolecular interactions induced by pressure reduce energy loss through nonradiative rotational relaxation channels, thereby enhancing the emission. Although significant emission enhancement was realized in a specified pressure range, the luminescence was suppressed under further pressurization, perhaps due to the *π*–*π* interactions, the formation of excimers and exciplexes, or an amorphous phase^54–56^.

However, the aforementioned studies explored structural regulation under pressure using a single wavelength or a series of excitation wavelengths over a narrow range. The changes in the molecular conformation, stacking mode, and intermolecular interactions could affect the absorption with changes in pressure, thus accompanying by the varied optimal excitation wavelength. Therefore, studying the emission behaviors using multiple excitation channels could lead to a more comprehensive exploration and thus a better understanding of the relationship among the molecular conformation, stacking mode, intermolecular interaction, and luminescence behavior under high pressures. However, only a few studies have focused on this aspect to date. As pressure-dependent changes in molecular conformation, stacking mode, and intermolecular interaction are unpredictable and unmanageable, most AIE molecules would not meet our experimental purpose. Recently, diphenyl-dibenzofulvene (DP-DBF) derivatives have consisted of intriguing AIE luminogens, which exhibited very interesting polymorphism-dependent emission^57–60^. Gu et al. also reported^61^ a special class of polymorphism-dependent luminescent AIE molecules with their emission characteristics regulated by modulating the molecular conformation and changing the stacking mode. Among them, the dihedral angles between the fluorene and benzene rings in di(*p*-methoxylphenyl)dibenzofulvene (referred to as FTPE in this study) were found to affect the degree of intermolecular *π*-conjugation, and in turn its emission properties. In addition to experimental studies, theoretical calculations revealed that the emission from FTPE differs according to the twist angles. Besides the restricted molecular rotation, the molecular conformation modulation, stacking mode transformation, and intermolecular interaction change caused by the aggregation microenvironment were also the main factors affecting the FTPE luminescence^62^. Therefore, we deduced that such a unique molecular group can be an ideal platform to explore the relationship between the molecular conformation, stacking mode, intermolecular interaction, and emission behavior under high pressures applied in multiple excitation channels.

In this study, we conducted a series of high-pressure photophysical experiments on FTPE single crystals to unveil the pressure-induced evolution of the aggregation microenvironment, which in turn can affect the molecular conformation, stacking mode, and intermolecular interaction. High-pressure angle-dispersive X-ray diffraction (ADXRD) and infrared (IR) studies indicated that the molecular conformation did not change obviously under low pressures, with the applied pressure mainly causing shortening of the intermolecular distance, leading to a rapid cell volume shrinkage. When the FTPE crystal was compressed to a certain extent, there was insufficient space for the molecules to maintain the prevailing molecular conformation. As a result, the molecular conformation changed largely, inducing a planar adjustment of the crystalline structure and tight molecular stacking, thereby leading to an isostructural phase transformation and enhanced intermolecular interactions. Furthermore, as changes in the molecular conformation, stacking mode, and intermolecular interaction could influence the energy level of the system, the emission behavior of the molecule could be adjusted by applying pressure. Under multiple excitation channels, three distinct emission behaviors were observed corresponding to the pressure-induced structural evolution, which is a meaningful phenomenon, but has rarely been observed before. This work not only provides an unconventional strategy for examining the structure-property relationship of FTPE under pressure-induced aggregation microenvironment evolution using multiple excitation channels, but also promotes the development of stimulus-responsive luminescent materials.

## Results

### Sample characterization under ambient and high pressure

**Fig. 1:**
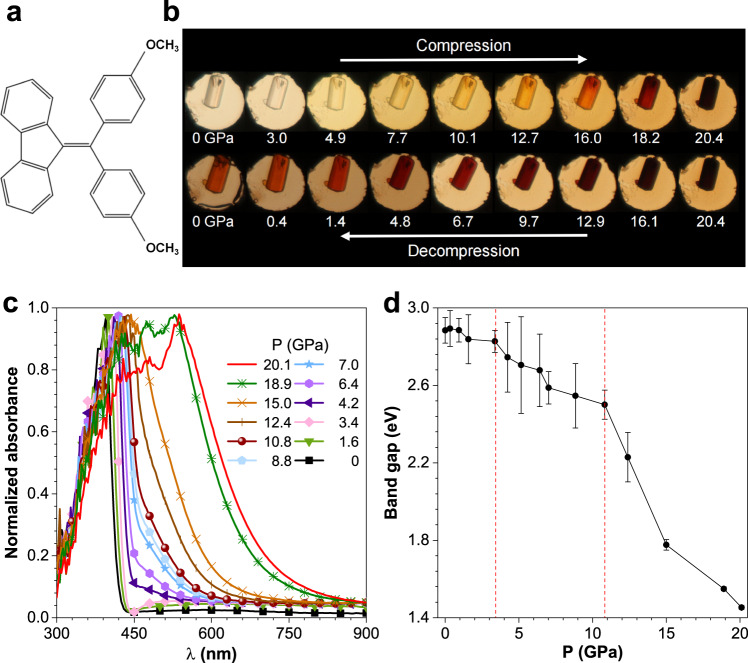
Absorption characterization of FTPE crystal under pressure. **a** Atomic structure of FTPE molecule. **b** Images of FTPE single crystal during compression and decompression processes (0 GPa indicates ambient pressure). In situ (**c**) UV–vis absorption spectra and (**d**) band gaps of FTPE at different pressures (P). The error bars are estimated according to the intercept and slope of the fit curve.

FTPE is a typical organic fluorophore with a monoclinic *C2* symmetry; its atomic structure is shown in Fig. 1a. In crystals, the FTPE molecules are arranged in a layered form along the *b*-axis. The molecular stacking of FTPE crystal is shown in Supplementary Fig. 1. In each layer, the molecules are linked via weak intermolecular interactions involving C–H···*π* interactions, but lack *π*–*π* interactions; that is, the fluorene and benzene rings of neighboring molecules are not parallel. Previous studies have shown that the molecular conformation of FTPE plays a crucial role in its luminescence behavior: the higher the coplanarity of the fluorene and benzene rings is, the higher the degree of intermolecular *π*-conjugation is. Besides, the stacking mode and intermolecular interactions also contribute to the luminescence properties. Accordingly, the absorption edge in the ultraviolet-visible (UV–vis) spectrum and emission maximum in the luminescence spectrum exhibit a red shift^61^. Figure 1b shows the images of an FTPE single crystal captured during compression and decompression processes. The crystal was pale yellow with good transparency at atmospheric pressure under daylight. During the compression process, the color changed to orange red and then dark red at 20.4 GPa. Upon releasing the pressure to atmospheric pressure, the FTPE single crystal sample did not recover its initial color, indicating that the color-changing phenomenon was not reversible. Previous studies have demonstrated that the color evolution during the compression process is accompanied by a red shift in the absorption edge^38,49,52^. Figure 1c shows the UV–vis absorption spectrum recorded under different pressures. In ambient conditions, the absorption edge of FTPE was found be at 395 nm, corresponding to a band gap of 2.88 eV, consistent with previous reports^61^. Details of the UV–vis absorption spectra under different pressures are shown in Supplementary Fig. 2. When the pressure was increased, the UV–vis absorption spectrum showed a clear red shift, as expected. Interestingly, a new absorption edge appeared between 450 and 550 nm at 4.2 GPa. At the same time, the shape of the absorption spectrum changed significantly, suggesting the most probable enhancement of the planarity of FTPE molecular conformation with higher conjugated degree and the changes of stacking mode and intermolecular interaction by increasing pressure^63^. When the pressure exceeded 12.4 GPa, the distinction between the original and new absorption edges began to blur. With further pressurization, new absorption peaks appeared at 474 nm and 531 nm. Figure 1d plots the change in the band gap (*E*_g_) with pressure, which was determined in *Tauc* coordinates method (Supplementary Fig. 3)^64^. Below 4.2 GPa, the band gap decreased only slightly from 2.88 to 2.82 eV. Between 4.2 and 10.8 GPa, the band gap decreased more rapidly from 2.82 to 2.50 eV. Further, when the pressure exceeded 10.8 GPa, the rate at which the band gap decreased became even faster, with the band gap decreasing from 2.50 to 1.45 eV. Previous studies have demonstrated that the red shift of the UV–vis spectrum could possibly be due to an increase in the degree of molecular conjugation, and that pressure possibly induced stacking structural transformation^50,63,65^.

### Structure evolution of FTPE under high pressure

**Fig. 2:**
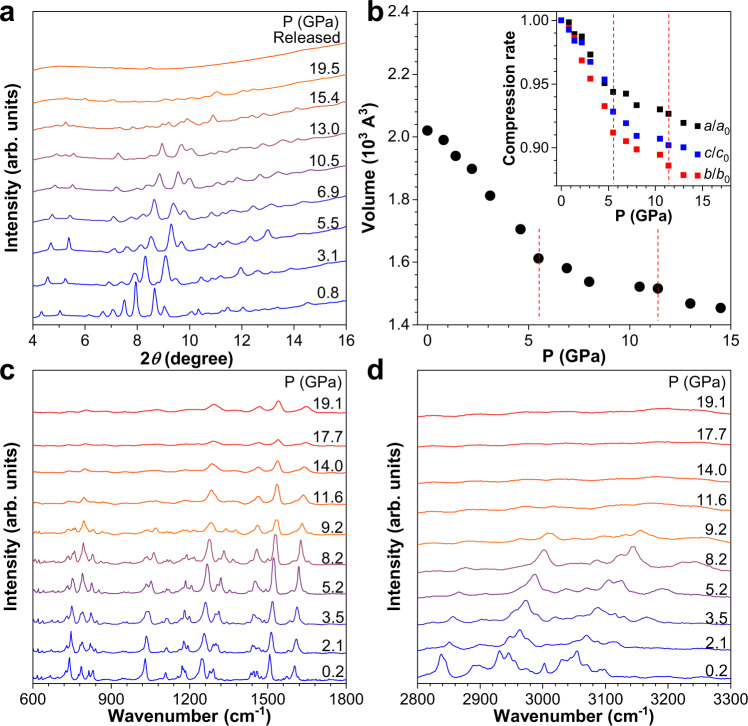
Structural evolution of FTPE crystal under high pressure. In situ (**a**) ADXRD patterns of the FTPE powder and (**b**) unit cell volume under pressure. The red dotted lines indicate isomorphic and crystalline-to-amorphous phase transitions. IR spectra of FTPE single crystal in (**c**) 600–1800 cm^−1^ and (**d**) 2800–3300 cm^−1^ ranges at different pressures in the range of 0.2–19.1 GPa. Inset of (**b**) shows the compression rate of the lattice constants (*a*/*a*_0_, *b*/*b*_*0*_, *c*/*c*_*0*_).

To investigate the influence of pressure on the structure of FTPE, we conducted in situ high-pressure ADXRD and IR spectral investigations in a diamond anvil cell (DAC) chamber at room temperature. Figure 2a shows the ADXRD data corresponding to discrete compression states (pressure range: 0.8 to 19.5 GPa), along with the data for the corresponding decompressed states at ambient pressure. At pressures below 13.0 GPa, the diffraction peaks clearly indicated that the sample was mainly in the crystal state, and all diffraction peaks shifted toward higher angles with increasing pressure because of the pressure-induced reduction in the unit cell volume and interplanar crystal spacing. However, at pressures larger than 13.0 GPa, the diffraction peaks became wider owing to diffuse scattering, suggesting that the amorphous phase was dominant. This result indicates that the sharp decrease in band gap (Fig. 1d) was due to amorphization. Moreover, upon releasing the pressure, the diffraction pattern did not correspond with the pattern obtained for the original sample, indicating that the process was irreversible. To further understand the relationship between the structure and pressure, we performed Le Bail refinement of the ADXRD patterns. Supplementary Fig. 4 and Supplementary Table 1 show the ADXRD patterns obtained under different pressures and the corresponding refinement results. The curves of the pressure-dependent lattice parameters and volume could be divided into three sections according to the change rate, as shown in Fig. 2b, indicative of the isostructural phase transition and amorphization of the sample. The pressure at which the isostructural phase transition occurred coincided with the pressure at which a new absorption edge emerged in the UV–vis absorption spectrum. Therefore, we deduced that, below 5.0 GPa, the lattice parameters and volume decreased rapidly owing to easy compression. Above 5.0 GPa, there was no more available interspace for further molecular packing. However, the molecular conformation continued to change in response to the pressure increase, leading to a higher elastic modulus and small pressure-dependent volume change. When the pressure was above 13 GPa, the amorphous component was dominant, consistent with the sharp decrease in the band gap. Simultaneously, the refinement results suggested that the compression of FTPE was anisotropic, with the *b*-axis being the most compressible axis. Combined with the molecular stacking of FTPE (see Supplementary Fig. 1), we inferred that the pressure rapidly shortened the intermolecular packing distance, at the same time parallelized the molecular conformation, which may increase the *π*–*π* overlapping degree of adjacent molecules, or even to form excimers^32^. In addition, the MALDI-TOF of FTPE at ambient pressure, released from 6.6 GPa, 11.5 GPa, and 19.5 GPa indicated that there would probably be no molecular polymerization production during the pressurization process (see Supplementary Fig. 5).

Further, in situ high-pressure IR experiments were conducted to obtain information on the chemical bonding and local structure. Figure 2c, d shows the IR spectra of the FTPE single crystal in the 600 to 3300 cm^−1^ range under different pressures. Materials Studio was used to analyze the IR vibrational modes under atmospheric pressure, and the results are shown in Supplementary Table 2^66^. By elevating the pressure, most of the IR peaks were blue-shifted indicating that the interatomic distances were decreased but in different extent^50,54,55^. Below 5.2 GPa, pressure mainly affected the stacking mode, causing a dominating reduction in cell volume, which would restrict the stretching vibration of molecules through intermolecular interactions, probably contributing to the obvious blue shift at high-wavenumber region above 2800 cm^−1^. The emergency of the new peak at 3165 cm^−1^ (3.5 GPa) may be attributed to the formation of intermolecular C–H···*π* interactions. At pressures higher than 5.2 GPa, the pressure mainly caused a change in the molecular conformation, because of which the blue shift of the low-wavenumber peaks corresponding to molecular rotation was more conspicuous as tight stacking would greatly hinder the aromatic rings to rotate freely^63^. The blue shift of the rotational modes became clearer after the compression-induced conformational change, and the shape of most IR peaks changed noticeably, suggesting that the applied pressure increased the strength of intermolecular interactions. Above 11.6 GPa, most IR peaks became broader and the stretching vibration signals weakened, whereas the characteristic peaks of the conformation-related rotational vibrations were still observed to some extent, indicating the emergence of an amorphous phase, consistent with the ADXRD data. Supplementary Fig. 6a–d shows the IR peaks of the sample pressurized to 4.9 and 11.5 GPa. As shown in Supplementary Fig. 6e, after the release of pressure, the IR peaks of the sample pressurized to 4.9 and 11.5 GPa were similar to those under atmospheric pressure, suggesting that the isostructural phase transition was reversible. However, following pressure release from 19.1 GPa the IR peaks of the sample were different from those of the original sample, consistent with the ADXRD data, indicating that the structural change was irreversible.

### Fluorescence measurements of FTPE under high pressure

**Fig. 3:**
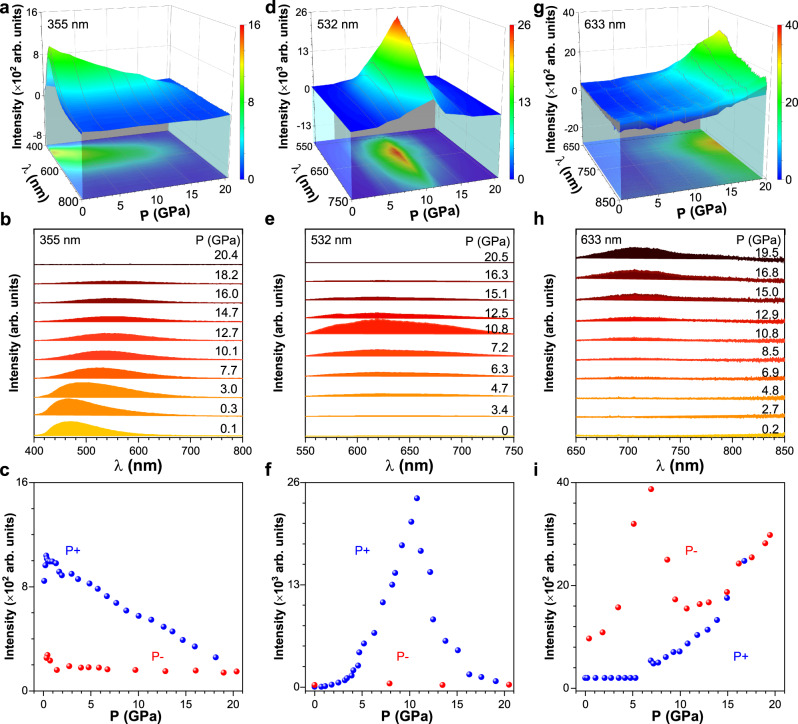
Fluorescence monitoring of FTPE crystal structural evolution under high pressure. Pressure-dependent 3D and 2D fluorescence spectra of FTPE single crystal during the compression process under laser excitation at (**a**, **b**) 355 nm, (**d**, **e**) 532 nm, and (**g**, **h**) 633 nm. Changes in the fluorescence intensity with pressure during compression and decompression processes under laser excitation at (**c**) 355 nm, (**f**) 532 nm, and (**i**) 633 nm. The color in (**a**, **d**, **g**) indicates the magnitude of fluorescence intensity. P+ and P- in (**c**, **f**, **i**) indicate the fluorescence during the compression and decompression, respectively.

To examine the influence of the stacking mode and molecular conformation on the emission properties, three excitation wavelengths were chosen based on the absorption data to explore the emission properties under high pressure. Figure 3 shows the pressure-dependent 3D and 2D fluorescence spectra of FTPE single crystal excited with 355, 532, and 633 nm lasers (the separated fluorescence spectra were displayed in Supplementary Figs. 7–9). Combined with the structural data, it could be inferred that the molecular movement was more restricted under pressure than that in the initial crystal state owing to the reduced distance between the molecules. Therefore, the energy was released in the form of radiative transition, resulting in an increase in the intensity of the emission obtained under 355 nm excitation below 0.3 GPa (Fig. 3a). At low pressures, a blue shift was observed in the high-wavenumber IR peaks related to cell volume, indicating that the pressure mainly shortened the intermolecular distance of adjacent molecules. Accordingly, with increasing pressure, the emission obtained under 355 nm excitation decreased in intensity, and there was a red shift of the emission maximum. Further increase in pressure led to a planar conformation, resulting in an increase in intramolecular conjugation^65^. At the same time, the decrease of intermolecular distance may favor the polarization effect on adjacent molecules, increase the intermolecular interaction, or drive the switch from crystalline state to amorphous state, which would reduce the energy of the lowest excited state, leading to a red shift in the fluorescent wavelength and a decrease in intensity^67,68^. Consequently, the emission showed an apparent red shift of the emission maximum, and the emission intensity decreased further due to increased conjugation and the transformation of the crystal state from its initial state to a state with planar conformation (Fig. 3b). When the sample became amorphous, the emission maximum did not shift with the pressure. Eventually, the emission band almost disappeared at 20.4 GPa due to the quite low quantum efficiency for the amorphous state and emission-detriment intermolecular interactions such as *π*–*π* interactions. Following the pressure release, the emission intensity did not return to the initial value, indicating that the conformational change was irreversible (Fig. 3c). Although the molecular conformation tended to recover to the initial one, the stacking mode had changed, and the molecules had rearranged. As a result, the transformation was irreversible. The pressure-dependent color change under 355 nm laser excitation is shown in Supplementary Fig. 10, along with the corresponding emission spectra.

Figure 3d, e shows the pressure-dependent fluorescence spectra obtained under 532 nm laser excitation over the pressure range of 0 to 20.5 GPa. According to the UV–vis spectra (Fig. 1c), the absorbance at ~532 nm increased marginally with pressure, and accordingly, the intensity of the emission under 532 nm laser excitation increased slowly during low pressure range. After the isostructural phase transition above 4–5 GPa, the absorbance increased abruptly with the appearance of a new absorption edge between 450 and 550 nm, resulting in a significant enhancement of the emission intensity (Fig. 3f). Therefore, these interesting emission behaviors under 532 nm laser excitation could be reasonably employed to investigate the isostructural phase transition within crystal under pressure. Notably, there was no obvious red or blue shift of the fluorescence band, different from the results obtained for most luminescent materials under high pressures^49,55^. As mentioned above, the fluorescence band underwent a red shift due to the pressure-induced conformational planarization, stacking mode change, and intermolecular interaction enhancement, as observed in the fluorescence spectra obtained under 355 nm laser excitation. Combined with the relationship of the emission intensity, it might be inferred that the molecules that were gradually turning planar might have been excited by the 532 nm laser. Therefore, there was a sharp increase in the emission intensity after the isostructural phase transition of FTPE, but the excited state energy may relax to the potential well to form an equilibrium state^69^ or the molecular conjugation could perhaps be unchanged, resulting in an almost unchanged emission maximum with increasing pressure. At pressures greater than 10.8 GPa, the fluorescence intensity decreased rapidly until the weakly luminescent state was reached at a pressure of 20.5 GPa due to the transformation of the highly emissive crystal to a weakly emissive amorphous phase^61^. Therefore, despite the gradual increase in the molecular absorbance with pressure, the emission intensity decreased. When the amorphous state dominated, the emission intensity decreased more slowly. Following the pressure release, the fluorescence characteristics did not revert to the initial ones, consistent with the results obtained under 355 nm laser excitation, indicating that the process was irreversible. Moreover, the pressure-dependent normalized lifetime spectra excited by the 532 nm laser and the calculated decay times of FTPE also presented the three distinct processes of 0.6–4.8 GPa, 4.8–10.1 GPa, and 10.1–19.0 GPa, as shown in Supplementary Fig. 11, which are well corresponding to the PL and X-ray diffraction results.

Similarly, when excited with the 633 nm laser under pressure, in addition to the absorbance behavior, the emission behavior was also completely different from those observed under other excitation wavelengths. According to the UV–vis results, the absorbance at ~633 nm was almost zero at low pressure, and there was no emission or weak emission with a constant pressure-dependent intensity below 5.3 GPa (Fig. 3g, h). After the isostructural phase transition, the intensity of the emission increased gradually. Further, the emission maximum did not shift with increasing pressure, as observed under 532 nm laser excitation. Figure 3i shows an abrupt increase in the emission intensity with pressure and a higher rate of the emission intensity increase after 12.9 GPa, which is the pressure corresponding to the emergence of the amorphous state according to the ADXRD results in Fig. 2a. In addition, the sample exhibited weak luminescence when the pressure was released. Combined with the pressure–structure data, it could be inferred that the fluorescence observed under the 633 nm laser excitation might be due to the amorphous phase generated under the compression force. The crystal exhibited no luminescence between 0 and 5.3 GPa under 633 nm laser excitation because the molecular structure was not in the amorphous phase in this pressure range. As the nonhydrostatic pressure was increased further, a part of the crystal phase turned amorphous, resulting in a gradual enhancement of the emission. Above 13.0 GPa, the structure gradually changed from a crystalline to amorphous phase, accompanied by an appreciable enhancement of the emission. These results evidently supported that 633 nm laser excitation channel could be used to monitor the pressure-dependent emission behavior of the amorphous state. Following the pressure release, the sample showed a faint glow, implying that the molecules could not revert to their initial state completely from the amorphous state after being pressurized to 19.5 GPa, consistent with the results obtained under 355 and 532 nm laser excitation. Such phenomenon motivated us to further monitor the fluorescence spectra of FTPE in amorphous state under different pressures through the sample prepared in situ after releasing from the pressure of about 20 GPa. As shown in Supplementary Fig. 12c, d, the emission intensity increased linearly under the pressure ranging from atmosphere pressure to about 20.0 GPa under 633 nm laser excitation. This fluorescence observed under the 633 nm laser excitation was assigned to the amorphous phase generated under compression, which was similar to the emission behavior under the pressure ranging from 12.5 GPa to 20.0 GPa in Fig. 3g–i and Supplementary Fig. 12a, b, further implying the transformation between the crystalline and amorphous states for FTPE. In addition, Supplementary Fig. 13 shows the pressure-dependent emission characteristics under 355, 532, and 633 nm laser excitations when the sample was pressurized up to ~10 GPa. During the compression and decompression processes, the emissive states excited by the 355 and 532 nm lasers were consistent with the initial state, whereas the emissive state excited by the 633 nm laser was not (Supplementary Fig. 13g–i), suggesting the possible transformation pressure between crystalline and amorphous states is about 10 GPa.

**Fig. 4:**
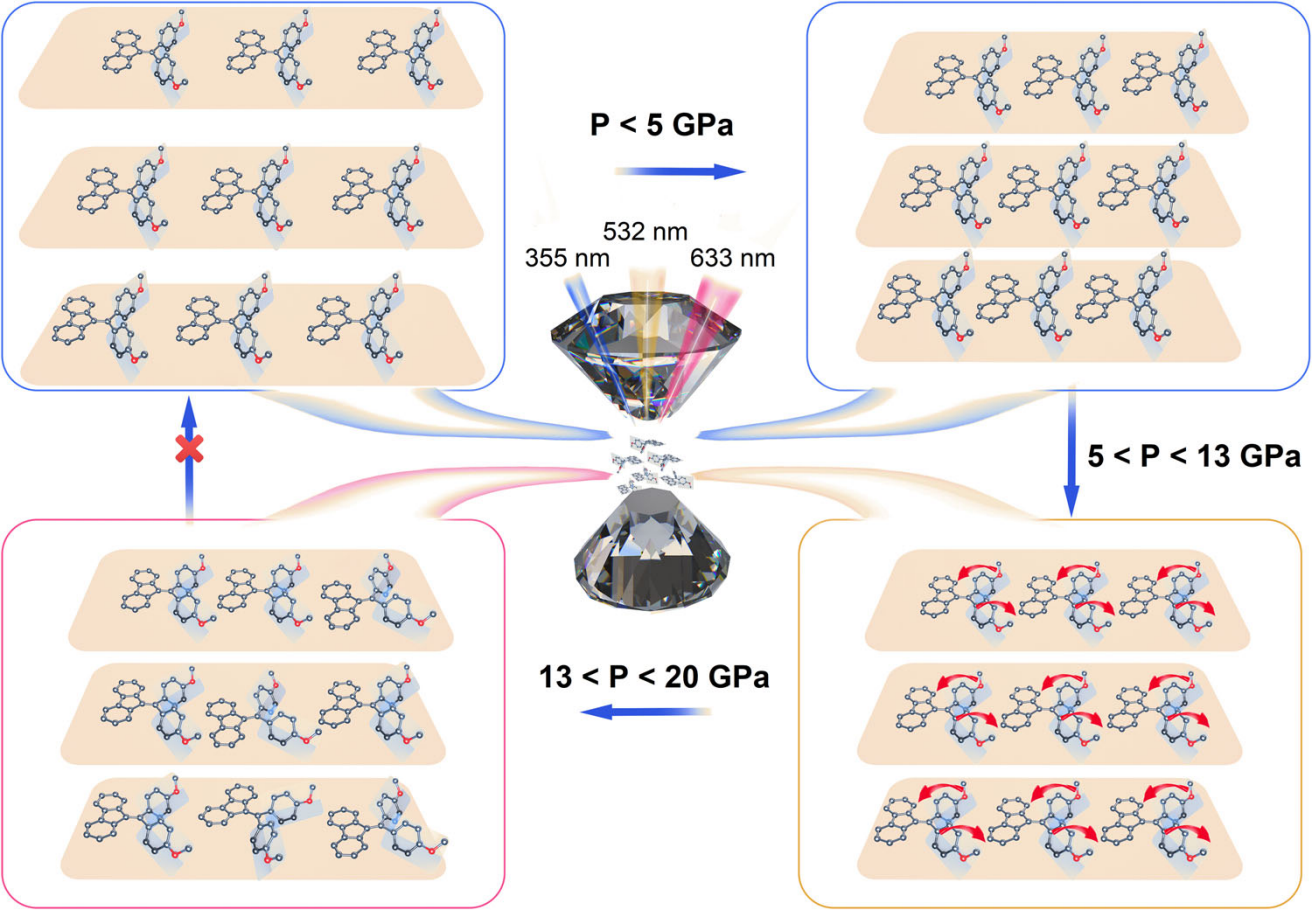
Schematic of FTPE crystal structural evolution under high pressure via multiple excitation channels. The entire compression process can be divided into three stages: (1) the pressure reduces the distance between the molecules, particularly along the *b*-axis, without obvious molecular conformation change, (2) owing to the limited space, the molecular conformation would adjust to be planar for adapting to the smaller space under pressure, and (3) a further compression leads to the amorphization of the structure.

Thus, by selecting three different laser excitation wavelengths, the changes in the molecular stacking and molecular conformation could be monitored, as shown in Fig. 4. Despite the slight modulation of molecular conformation, the closer stacking mode, corresponding to the change of cell volume, played a major role below ~5 GPa. That is, the blue shift of the low-wavenumber IR peaks associated with the molecular conformation was not obvious, while the sensitivity of the high-wavenumber peaks to volume change was apparent. Meanwhile, the decrease in the distance between the molecules in the crystal might lead to an increase in the intermolecular interaction, polarization effect on adjacent molecules or the amorphous evolution, and the energy was dissipated through nonradiative pathways. Thus, the emission intensity decreased gradually, accompanied by a red shift of the emission maximum under 355 nm laser excitation. In the meanwhile, the continuous red shift in PL emission and the decreasing band gaps calculated by the UV–vis absorbance reflected the planarity tendency of molecules and conjugation-benefit intermolecular interactions that enhanced the intramolecular and intermolecular conjugation. When the isostructural phase transition occurred, the molecules flattened gradually under pressure, resulting in a blue shift of the low-wavenumber IR peaks associated with the conformation and a readily observable change in the peak shapes. The planarization of the molecular conformation causes a reduction in the number of molecules in the initial conformation and an increase in the number of molecules in the crystal state with planarized molecules. Such a numeric correlation gives rise to an increase in the fluorescence under 532 nm excitation until the appearance of amorphous. What is remarkable in the pressure-dependent fluorescence under 532 nm excitation is that the emission intensity suddenly increased at an expanding rate above 5 GPa, pointing to another factor that led to the enhancement of the intensity. The effect of restriction of intramolecular motions was considered as a result of the compression of molecular bonds and the enhancement of the intermolecular interactions those were observed in emergency of possible C–H···*π* at 3.5 GPa in the infrared spectra. Upon increasing the pressure, the molecular state gradually changed from a highly emissive crystal state into a weakly emissive amorphous state^61^. Therefore, despite the increase in absorption with the pressure, the emission under 532 nm excitation decreased rapidly above 10.8 GPa. However, the 633 nm laser could be used to monitor the emission of the amorphous state. Under excitation by 633 nm laser, the emission of amorphous state could be observed to increase with the pressure above 5 GPa, while there is almost no emission before ~5 GPa might be ascribed to the difficulty of transformation between crystalline and amorphous states under such low pressure. It has been proved that the amorphous state was unable to return to the crystalline state by merely removing the pressure, with no reversibility of fluorescence intensity and wavelength, as well as the IR absorbance, UV–vis absorbance, and ADXRD.

## Discussion

The significant evolution of the aggregation microenvironment involving changes in the molecular conformation, stacking mode, and intermolecular interaction was successfully monitored using multiple excitation channels. By analyzing the variations in the ADXRD and IR spectral features under high pressure, we determined that the applied pressure affected the cell volume before the isostructural phase transition occurred. The high-wavenumber IR peaks sensitive to volume change shifted noticeably, while the peak positions and shapes of low-wavenumber IR peaks mainly related to the molecular conformation essentially remained unobvious change. When the pressure was higher than 5 GPa, the peak positions and shapes of the low-wavenumber IR peaks related to the molecular conformation changed significantly, indicating that the pressure mainly affected the molecular conformation. The increase in the number of planar molecules resulted in a rapid increase in the emission intensity under 532 nm excitation. A further increase in pressure caused a structural change from the highly emissive crystal state to a weakly emissive amorphous state. Under 532 nm excitation, the emission intensity decreased rapidly, while the emission characteristic observed under 633 nm excitation changed from no emission to weak emission. When the amorphous state was predominant, the emission under 532 nm excitation was low, but the emission under 633 nm excitation increased rapidly. There was no interference of the emission behaviors under the three excitation wavelengths. Rather, the emission characteristics provided clear evidence of the isostructural phase transition and crystal-to-amorphous state transformation, which has rarely been realized before. This method is unprecedented and different from other previously reported approaches, and could provide vivid information on the molecular conformation, stacking mode and evolution of the phase (crystal to amorphous state) under applied pressure. This study provides an unconventional strategy to better understand the relationship between molecular conformation, stacking mode, and luminescence behavior, which is required for the design of stimulus-responsive organic light-emitting materials, according to the desired application.

## Methods

### High pressure generation

High-pressure experiments were performed using a DAC device (see Supplementary Fig. 14). Diamond anvils with a 300 μm diameter culet were used to generate high pressure. A T301 stainless steel gasket with initial thickness of 200 μm was preinserted to a thickness of 40 μm, and then a 150 μm diameter hole was drilled at the center of the indentation using a laser drilling machine to serve as a sample chamber. Small ruby balls were inserted into the sample cavity for in situ pressure calibration according to the R1 ruby fluorescence method^70^. Silicone oil was used as a pressure-transmitting medium (PTM) for optical absorption, PL, and ADXRD experiments. Potassium bromide was also used as a PTM in IR studies. In the optical absorption, PL, and IR experiments, type-IIa diamond anvils with a high transmittance were used to prevent the influence of diamond absorption and fluorescence. All experiments were performed at room temperature. Schematic diagram of the experimental setup was shown in Supplementary Fig. 15.

### Absorption studies

In situ absorption spectroscopy was performed using a UV–vis absorption spectrophotometer (Ocean Optics DH-2000-BAL) with a response time of 1 s. The wavelength ranged from 300 to 900 nm.

### ADXRD measurements

In situ high-pressure ADXRD experiments with an X-ray wavelength of 0.6199 Å were conducted at beamline BL15U1 in Shanghai Synchrotron Radiation Facility (SSRF) and beamline 4W2 in Beijing Synchrotron Radiation Facility (BSRF). The distance between the sample and detector was calibrated using a cerium (IV) oxide standard. The ADXRD patterns were collected for 60 s at each pressure, and the focused beam size was 3 μm. The ADXRD data were recorded using a Mar-165 charge-coupled device (CCD) detector and integrated into one-dimensional ADXRD patterns using the FIT2D program. The in situ ADXRD patterns were fitted using the Le Bail method implemented in the GSAS & EXPGUI software.

### IR measurements

The in situ high-pressure IR spectra were collected with a Bruker Vertex 70 v using a liquid nitrogen-cooled detector with a HYPERION 2000 microscope. A globar was used as a conventional light source. An aperture (50 × 50 μm^2^) was used to collect spectra in the transmission mode over the wavenumber range of 600–3300 cm^−1^ with a resolution of 2 cm^−1^.

### Computational details

Theoretical IR spectra were obtained using Materials Studio. We performed the analysis using density functional theory with the Perdew–Burke–Ernzerhof generalized gradient approximation using the Cambridge Serial Total Energy Package code. A norm-conserving pseudopotential with a plane-wave cutoff energy of 750 eV was used in the IR spectral calculations; a k-point separation of 0.07 Å^−1^ was used to generate k-points.

### Fluorescence experiments

High-pressure fluorescence measurements under 355 nm laser excitation were performed with an Ocean Optics QE65000 spectrometer with an inverted fluorescence microscope in reflection mode. The PL micrographs of the crystal under the excitation of 355 nm laser were acquired using a camera (Canon Eos 5D mark II) equipped with a microscope (Eclipse TI-U, Nikon). The camera captured photographs under consistent conditions with a constant exposure time and intensity. The high-pressure fluorescence measurements under the excitation of 532 and 633 nm lasers were performed using a confocal Raman micro-spectrometer: LabRAM HR Evolution, Horiba Scientific (see Supplementary Fig. 16). A diode-pumped solid-state laser was employed as the excitation light source with a focused 2.5 μm diameter laser beam. The signals of the fluorescence spectra were dispersed by a 600 g/mm^−1^ grating; the data collection time was 1 s.

### Time-resolved photoluminescence measurements

In situ high-pressure time-resolved PL measurements were performed with a time-correlated single photon counting (TCSPC, Becker & Hickl GmbH) coupled to a microscope (BX51M, Olympus). To generate the pump pulse with wavelength centred at 532 nm, the fundamental 800 nm pulse from a Titanium Sapphire mode lock laser (150 fs, 78 MHz, Coherent Mira-900D), was used to pump an optical parametric oscillator (OPO). The excitation laser power was maintained at approximately 5 μW to avoid overheating during measurements. The spectra for comparison were collected under the same conditions if there were no special instructions. The lifetime was obtained by deconvoluting the instrument response function (IRF) from the measured tr-PL data using the formula:

1 $${{{{{\rm{Y}}}}}}\left(t\right)={{{{{\rm{F}}}}}}\left(t\right)\otimes {{{{{\rm{IRF}}}}}}\left(t\right)={A}_{i}\,{{\exp }}\left(-t/{\tau }_{i}\right)\otimes A\,{{\exp }}\left(-{t}^{2}/{\sigma }^{2}\right)\\=\frac{\sqrt{\pi }}{2}{A}_{i}A\sigma \,{{\exp }}\left(-\frac{t}{{\tau }_{i}}+\frac{{\sigma }^{2}}{4{{\tau }_{i}}^{2}}\right)\left[1-{{{{{\rm{erf}}}}}}\left(\frac{\sigma }{2{\tau }_{i}}-\frac{t}{\sigma }\right)\right]$$

## Supplementary information

Supplementary Information

## Data Availability

Data supporting the results of this work are available within this paper or its Supplementary Information and are also available upon request from the corresponding author. The source data of FTPE CIF data, Figs. 1c, d, 2a–d, 3a–i, and Supplementary Figs. 2–9, 11–13 are provided in Source Data file. Source data are provided with this paper.

## Source data

Source Data
