# Supplementary material for: Fluorescence-based monitoring of the pressure-induced aggregation microenvironment evolution for an AIEgen under multiple excitation channels
Source: Nat Commun. 2022 Sep 6;13:5234. doi: 10.1038/s41467-022-32968-9 (PMC9448794; doi:10.1038/s41467-022-32968-9)
Supplement: Supplementary file 1 — Supplementary Information [file 41467_2022_32968_MOESM1_ESM.pdf]

Supplementary Information

Fluorescence-based monitoring of the pressure-induced aggregation microenvironment evolution for an AIEgen under multiple excitation channels

Shuang Tong[1, 2, 3]†, Jianhong Dai[2, 4]†, Jiangman Sun[3]†, Yuanyuan Liu[1], Xiaoli Ma[2], Zhehong Liu[2], Teng Ma[1,2], Jiao Tan[2], Zhen Yao[1], Shanmin Wang[4], Haiyan Zheng[5], Kai Wang[1], Fang Hong[2, 6], Xiaohui Yu[2, 6]+, Chunxiao Gao[1]+, and XingguiGu[3, 7]+

[1] State Key Laboratory of Superhard Materials, Jilin University, Changchun, 130012, China

[2] Beijing National Laboratory for Condensed Matter Physics, Institute of Physics,

Chinese Academy of Sciences, Beijing, 100190, China

[3] Beijing Advanced Innovation Center for Soft Matter Science and Engineering,

College of Materials Science and Engineering, Beijing University of Chemical Technology, Beijing,

100029, China

[4] Department of Materials Science and Engineering, Southern University of Science and Technology,

Shenzhen, 518055, China

[5] Center for High Pressure Science and Technology Advanced Research, Beijing, 100094, China

[6] Songshan Lake Materials Laboratory, Dongguan, Guangdong, 523808, China

[7] Beijing National Laboratory for Molecular Sciences, Beijing 100190, China

†These authors contributed equally to this work.

+Correspondence should be addressed to Xiaohui Yu (yuxh@iphy.ac.cn) or Chunxiao Gao (gaocx@jlu.edu.cn) or Xinggui Gu (guxinggui@mail.buct.edu.cn).

List of Contents

1. Supplementary Figures

Supplementary Figure 1. The molecular stacks within the crystal of FTPE.

Supplementary Figure 2. Pressure-dependent UV–vis absorption spectra of FTPE single crystal.

Supplementary Figure 3. The energy band gap of FTPE at 0 GPa.

Supplementary Figure 4. XRD patterns and refinement results of FTPE at different pressures.

Supplementary Figure 5. MALDI-TOF characterization.

Supplementary Figure 6. IR spectra of FTPE released from different pressure.

Supplementary Figure 7. Pressure-dependent fluorescence spectra of FTPE single crystal during the compression process under laser excitation at 355 nm.

Supplementary Figure 8. Pressure-dependent fluorescence spectra of FTPE single crystal during the compression process under laser excitation at 532 nm.

Supplementary Figure 9. Pressure-dependent fluorescence spectra of FTPE single crystal during the compression process under laser excitation at 633 nm.

Supplementary Figure 10. The daylight and fluorescent images of FTPE single crystal during compression and decompression under daylight and laser excitation at 355 nm.

Supplementary Figure 11. Time-resolved photoluminescence measurements.

Supplementary Figure 12. The fluorescence spectra of amorphous FTPE under different pressures.

Supplementary Figure 13. Fluorescence characteristics of FTPE during the compression.

Supplementary Figure 14. The physical photos of DAC.

Supplementary Figure 15. Schematic diagram of the experimental setup and the sample assembly.

Supplementary Figure 16. The photo of the DAC device during the fluorescence spectra collection.

2. Supplementary Tables

Supplementary Table 1. Refined Structural Parameters of the FTPE under high pressure.

Supplementary Table 2. IR modes of FTPE at 0.2 GPa and the calculated modes by Material Studio under atmospheric pressure.

1. Supplementary Figures

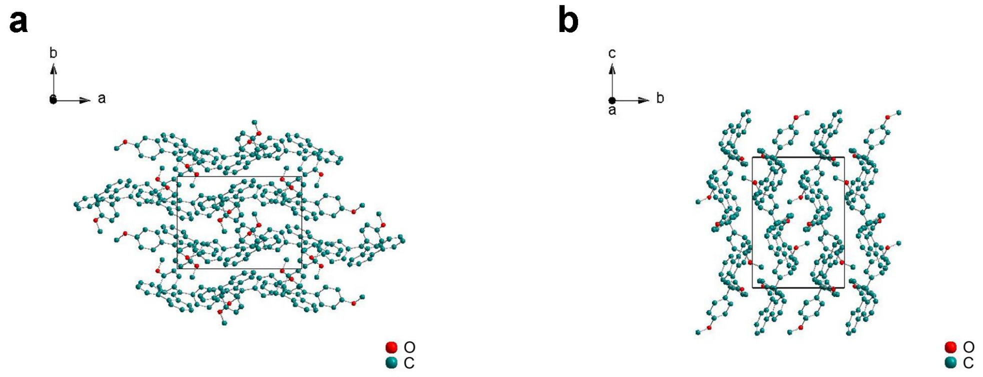

Supplementary Figure 1. The molecular stacks within the crystal of FTPE. (a) Views of the stacks running along *a*-axis. (**b**) Views of the stacks running along *c*-axis.

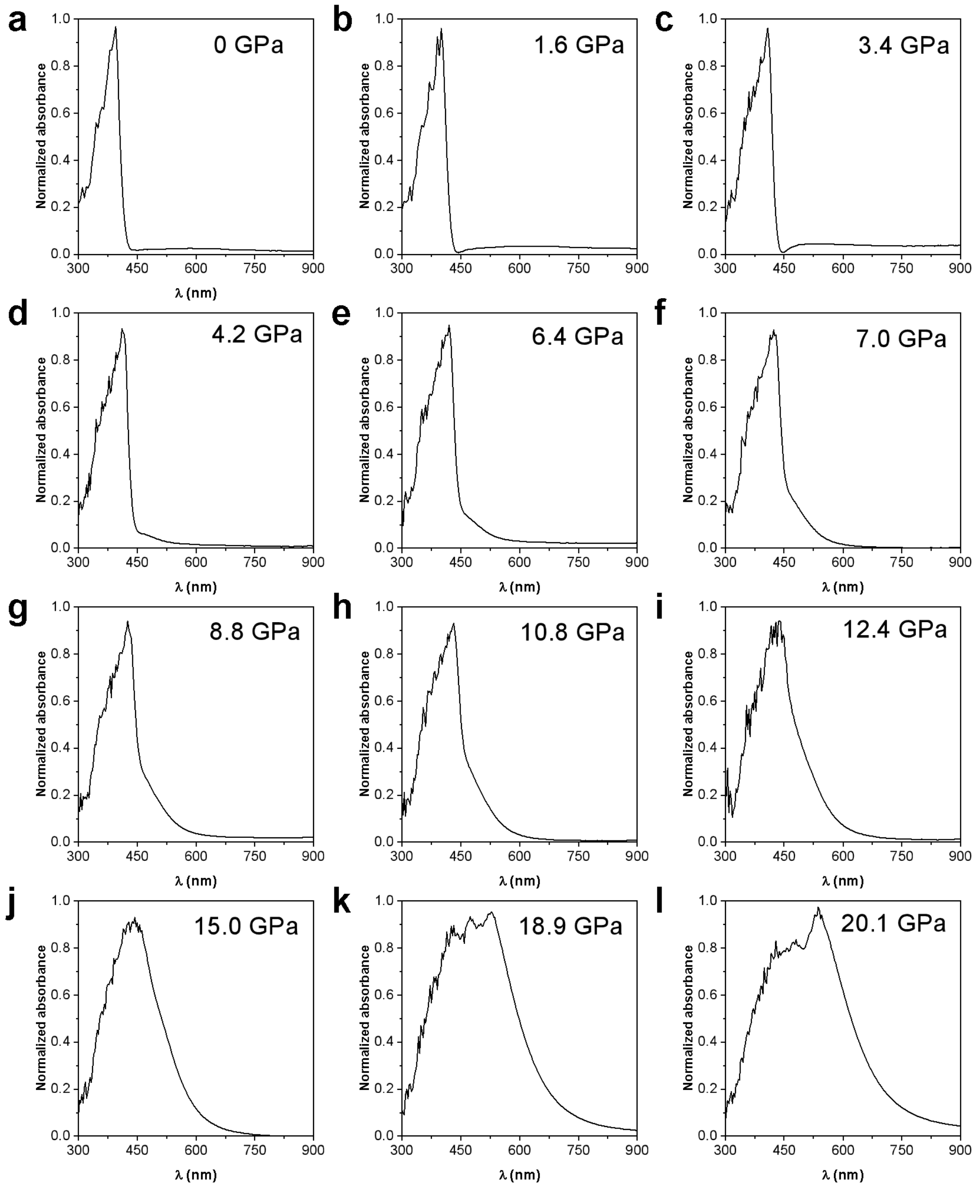

Supplementary Figure 2. Pressure-dependent UV–vis absorption spectra of FTPE single crystal. UV–vis absorption spectra of FTPE single crystal at (**a**) 0 GPa, (**b**) 1.6 GPa, (**c**) 3.4 GPa, (**d**) 4.2 GPa, (**e**) 6.4 GPa, (**f**) 7.0 GPa, (**g**) 8.8 GPa, (**h**) 10.8 GPa, (**i**) 12.4 GPa, (**j**) 15.0 GPa, (**k**) 18.9 GPa, and (**l**) 20.1 GPa. The absorption edge showed a clear red shift under high pressure. A new edge appeared between 450 and 550 nm at 4.2 GPa and new absorption peaks appeared at 474 nm and 531 nm after 18.9 GPa.

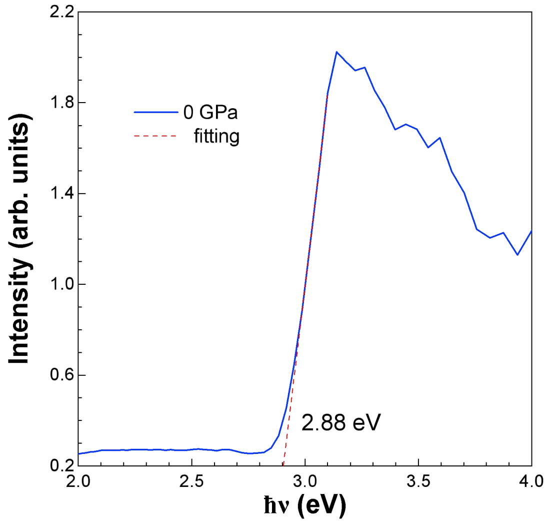

Supplementary Figure 3. The energy band gap of FTPE at 0 GPa. Method of band gap energy (E_g) determination from the *Tauc* plot. The linear part of the plot is extrapolated to the *x*-axis.

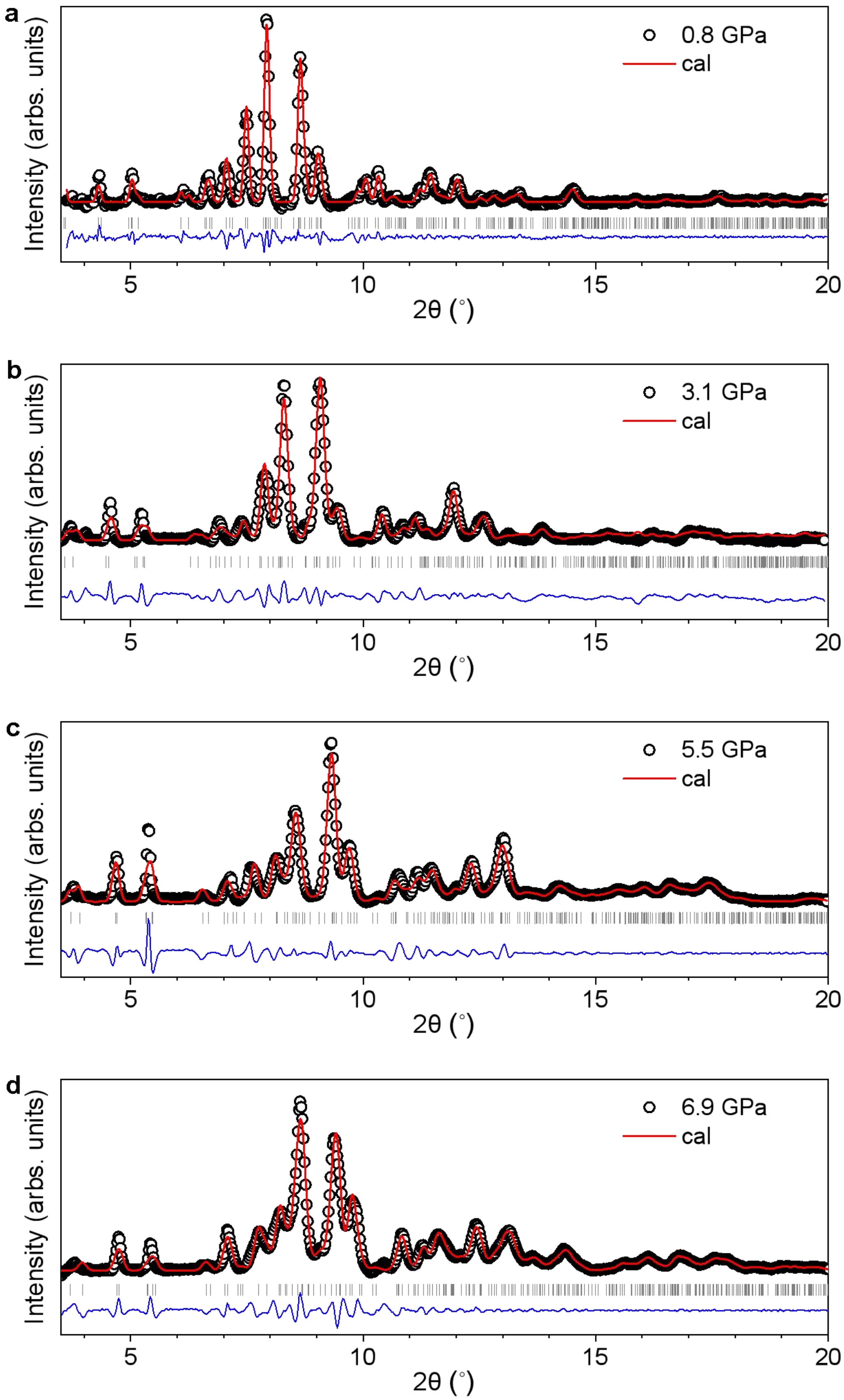
a
0.8 GPa
cal
b
3.1 GPa
cal
c
5.5 GPa
cal
d
6.9 GPa
cal
Intensity (arbs. units)
2θ (°)
5
10
15
20

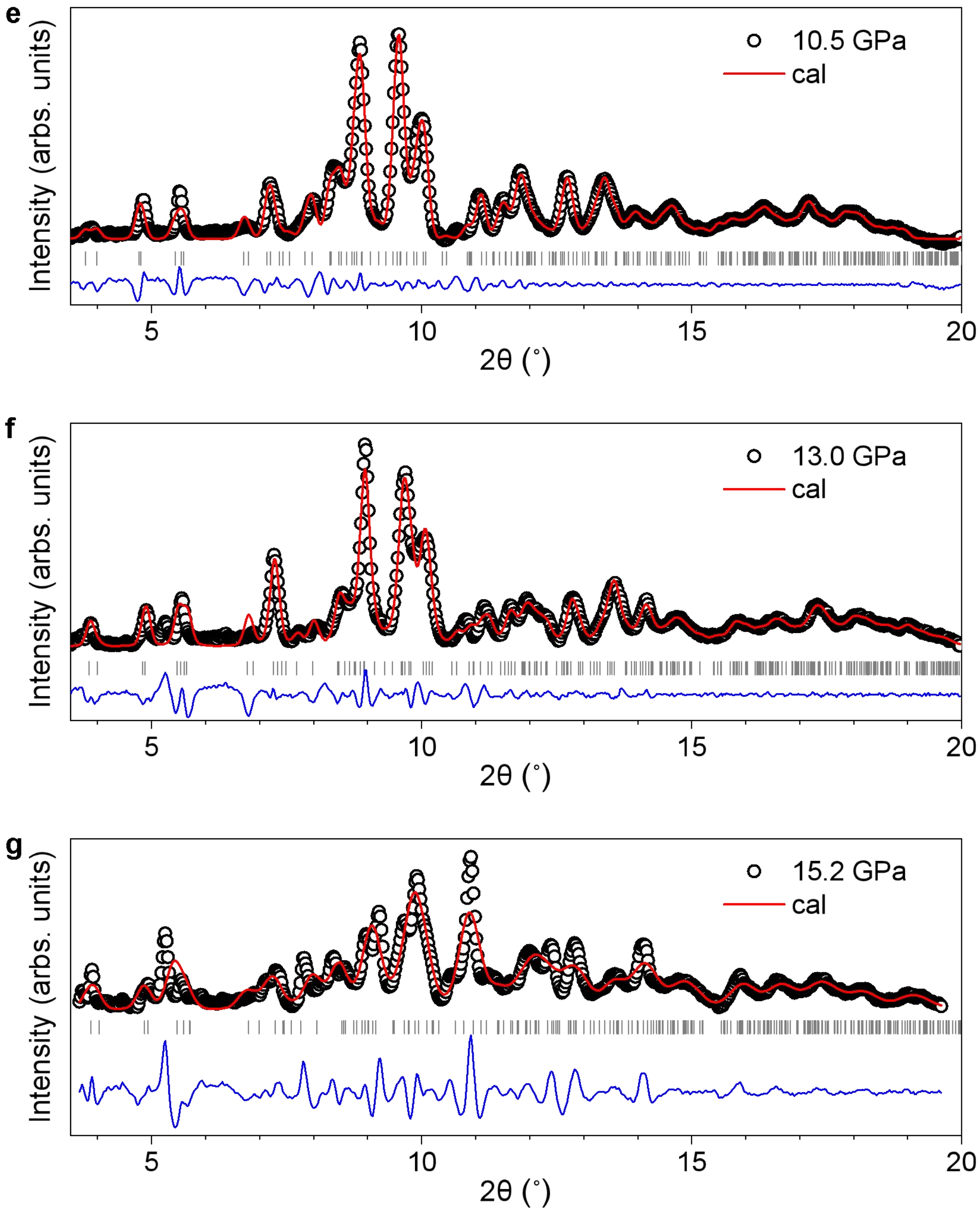

Supplementary Figure 4. XRD patterns and refinement results of FTPE at different pressures. The observed data (black circle), calculated (red line), and difference (blue line) values at (**a**) 0.8 GPa, (**b**) 3.8 GPa, (**c**) 5.5 GPa, (**d**) 6.9 GPa, (**e**) 10.5 GPa, (**f**) 13.0 GPa, and (**g**) 15.2 GPa are shown. The ticks indicate the allowed Bragg reflections for the $P2_1/n$ space group.

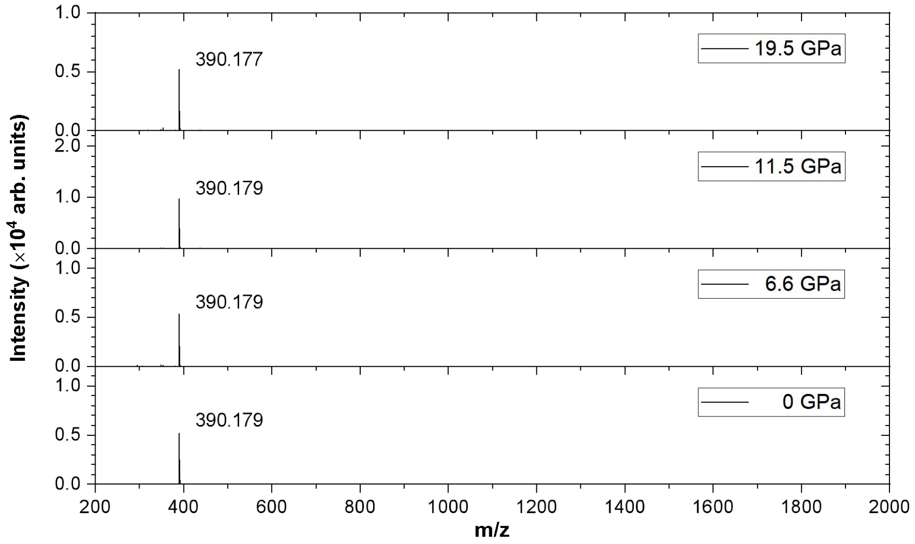

Supplementary Figure 5. MALDI-TOF characterization. The mass of FTPE was recorded at about 390.179 under 0 GPa, which did not change after releasing from 6.6 GPa, 11.5 GPa, and 19.5 GPa. Thus, there would probably be no molecular polymerization production during the pressurization process.

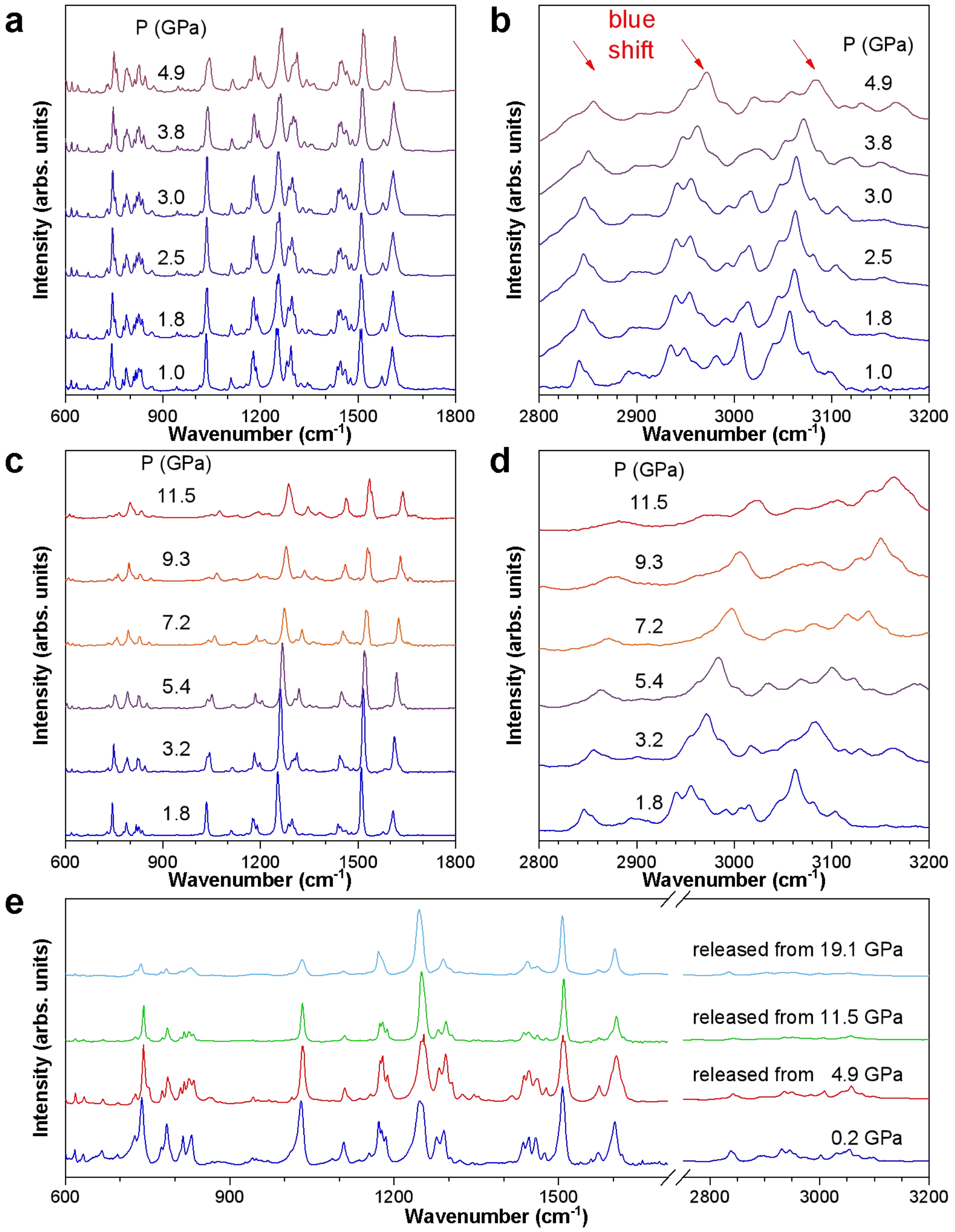

Supplementary Figure 6. IR spectra of FTPE released from different pressure. The IR spectra of FTPE single crystal in (**a**) 600–1800 cm^{-1} and (**b**) 2800–3300 cm^{-1} ranges at different pressures in the range of 1.0–4.9 GPa. IR spectra of FTPE single crystal in (**c**) 600–1800 cm^{-1} and (**d**) 2800–3300 cm^{-1} ranges at different pressures in the range of 1.8–11.5 GPa. Red arrows show an obvious blue shift at 4.9 GPa. (**e**) IR spectra of FTPE single crystal at 0.2 GPa and released from 4.9 GPa, 11.5 GPa, and 19.1 GPa.

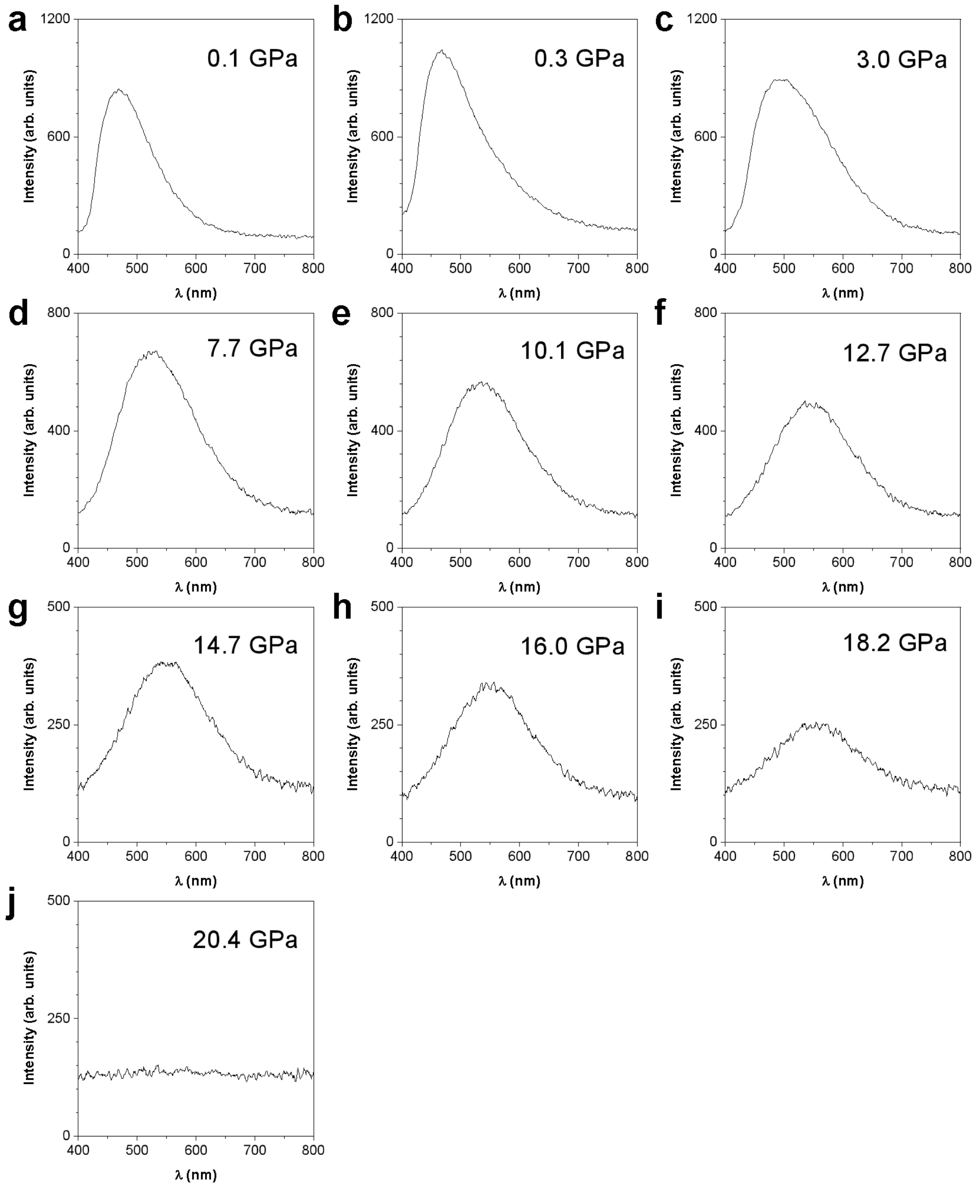

Supplementary Figure 7. Pressure-dependent fluorescence spectra of FTPE single crystal during the compression process under laser excitation at 355 nm. The fluorescence spectra of FTPE single crystal at (**a**) 0.1 GPa, (**b**) 0.3 GPa, (**c**) 3.0 GPa, (**d**) 7.7 GPa, (**e**) 10.1 GPa, (**f**) 12.7 GPa, (**g**) 14.7 GPa, (**h**) 16.0 GPa, (**i**) 18.2 GPa, and (**j**) 20.4 GPa. The fluorescence spectra increased with a blue shift when the sample was pressurized up to ~0.3 GPa and decreased with a red shift when the sample was pressurized up to ~10.1 GPa. Eventually, the fluorescence spectra almost disappeared at 20.4 GPa with no shift.

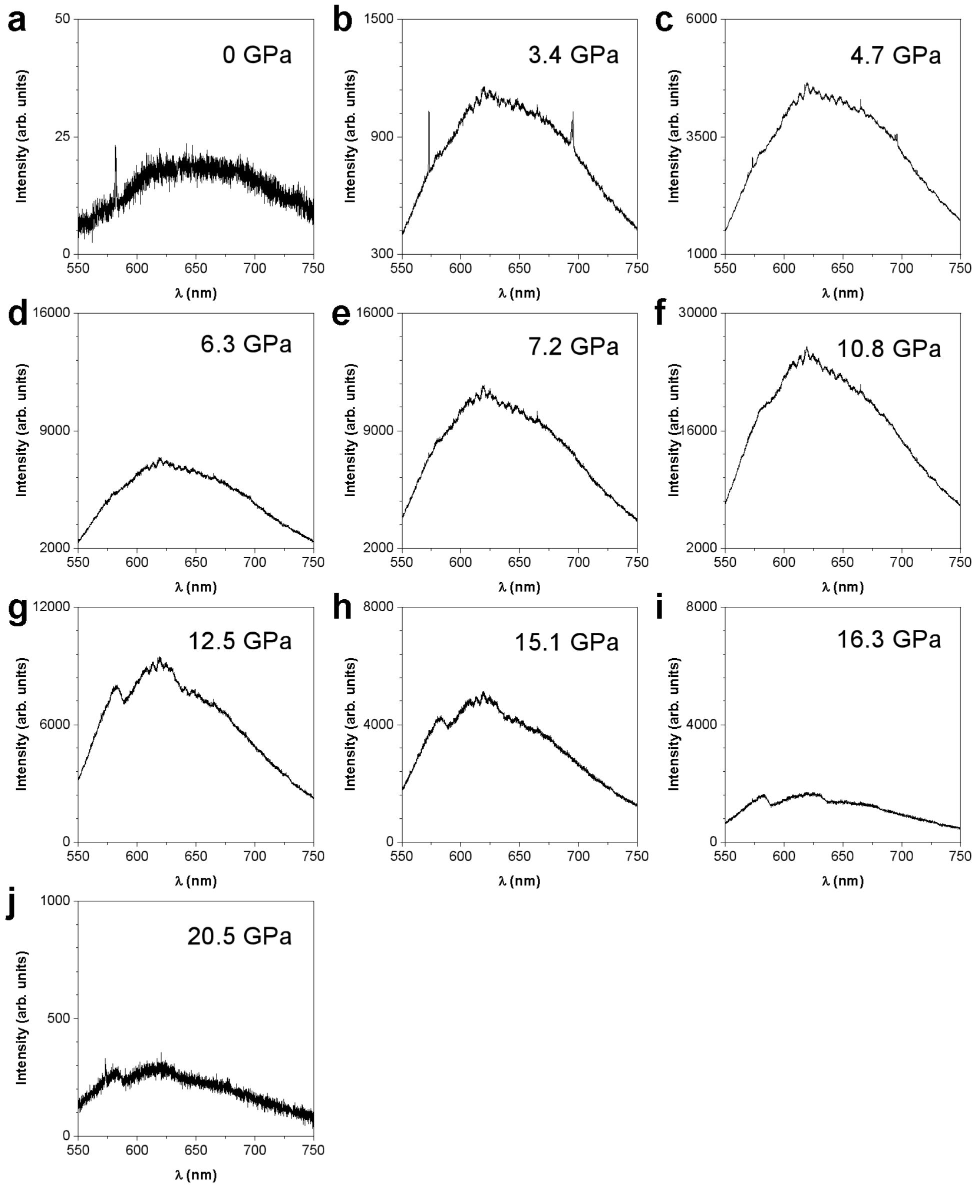

Supplementary Figure 8. Pressure-dependent fluorescence spectra of FTPE single crystal during the compression process under laser excitation at 532 nm. The fluorescence spectra of FTPE single crystal at (**a**) 0.1 GPa, (**b**) 3.4 GPa, (**c**) 4.7 GPa, (**d**) 6.3 GPa, (**e**) 7.2 GPa, (**f**) 10.8 GPa, (**g**) 12.5 GPa, (**h**) 15.1 GPa, (**i**) 16.3 GPa, and (**j**) 20.5 GPa. The fluorescence intensity has increased in the range of 0–10.8 GPa and decreased in the range of 10.8–20.5 GPa. There was no obvious red or blue shift of the fluorescence band.

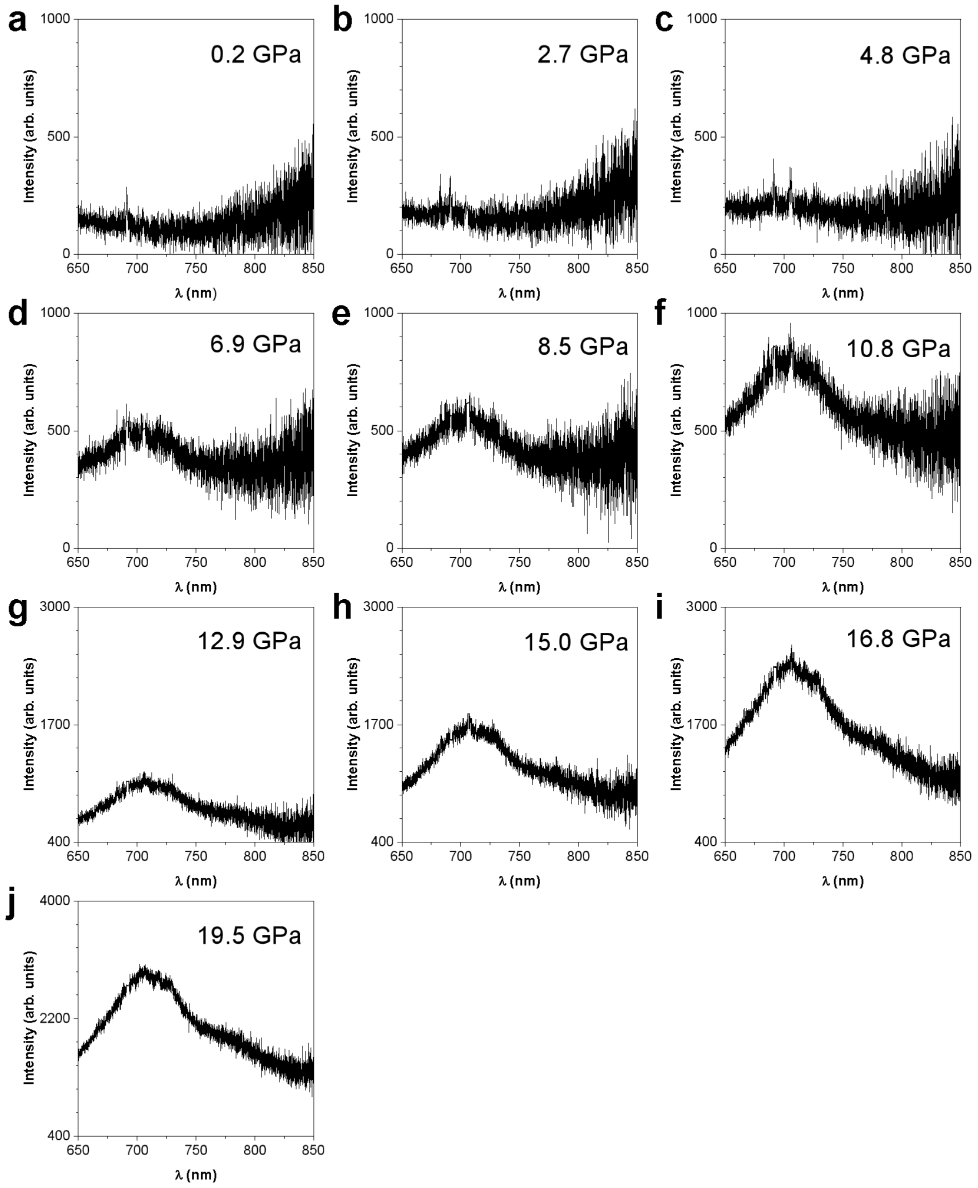

Supplementary Figure 9. Pressure-dependent fluorescence spectra of FTPE single crystal during the compression process under laser excitation at 633 nm. The fluorescence spectra of FTPE single crystal at (**a**) 0.2 GPa, (**b**) 2.7 GPa, (**c**) 4.8 GPa, (**d**) 6.9 GPa, (**e**) 8.5 GPa, (**f**) 10.8 GPa, (**g**) 12.9 GPa, (**h**) 15.0 GPa, (**i**) 16.8 GPa, and (**j**) 19.5 GPa. The fluorescence intensity has remained unchanged in the range of 0–4.8 GPa and increased in the range of 6.9–19.5 GPa. There was no obvious red or blue shift of the fluorescence band.

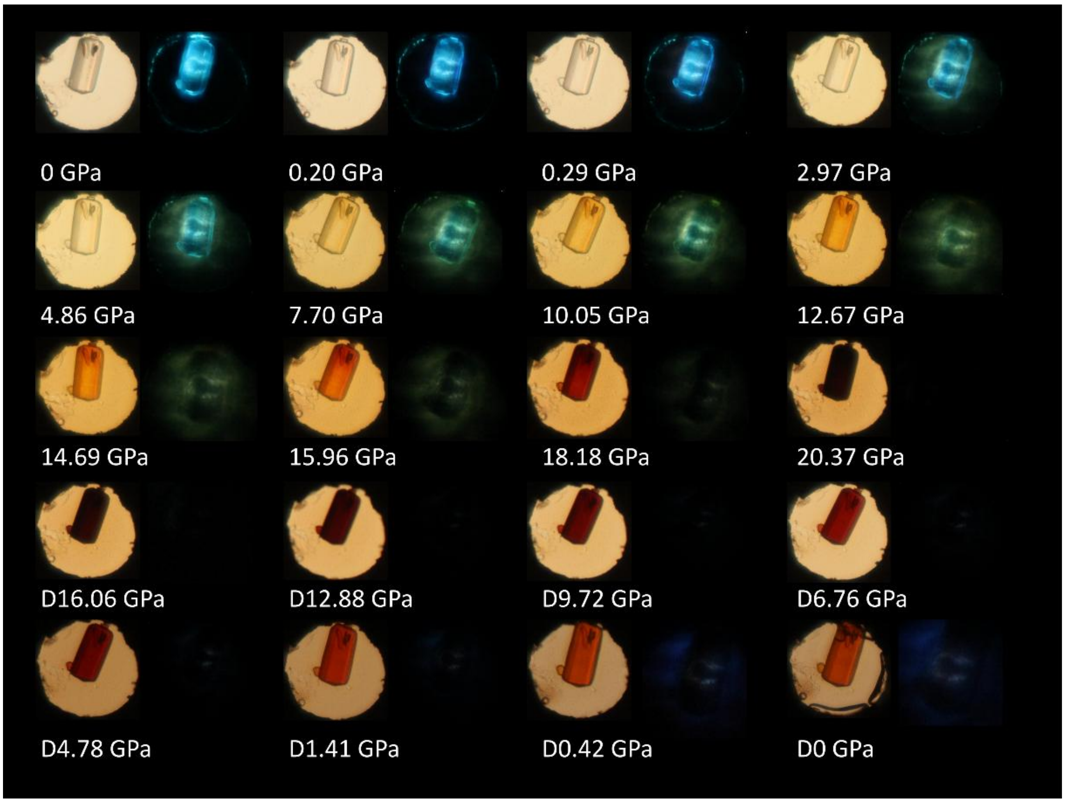

Supplementary Figure 10. The daylight and fluorescent images of FTPE single crystal during compression and decompression under daylight and laser excitation at 355 nm. Under daylight, the crystal was pale yellow with good transparency at 0 GPa and changed to orange red and dark red in the range of 0.2–20.37 GPa.

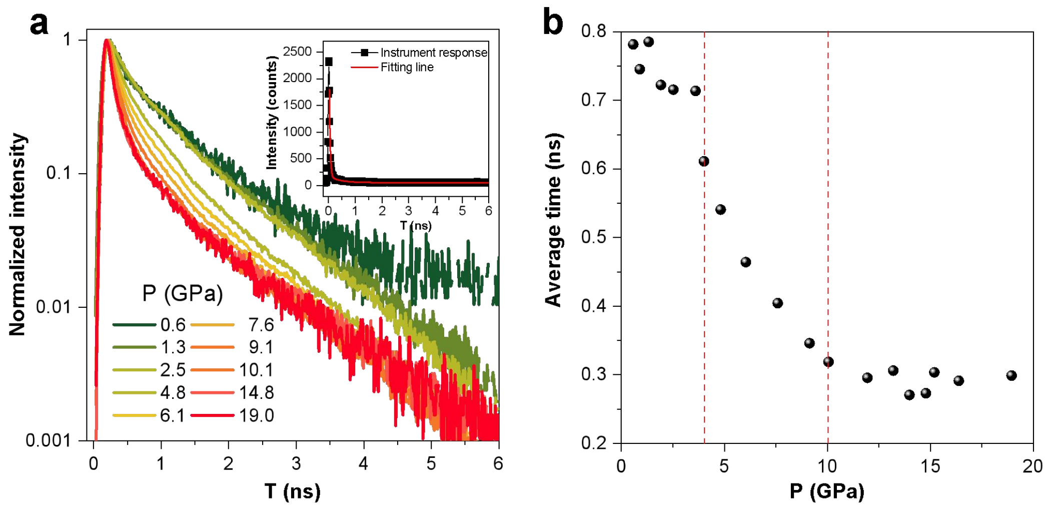

Supplementary Figure 11. Time-resolved photoluminescence measurements. (**a**) The normalized lifetime spectra during the pressurization and (**b**) the plot of average lifetime against pressure.

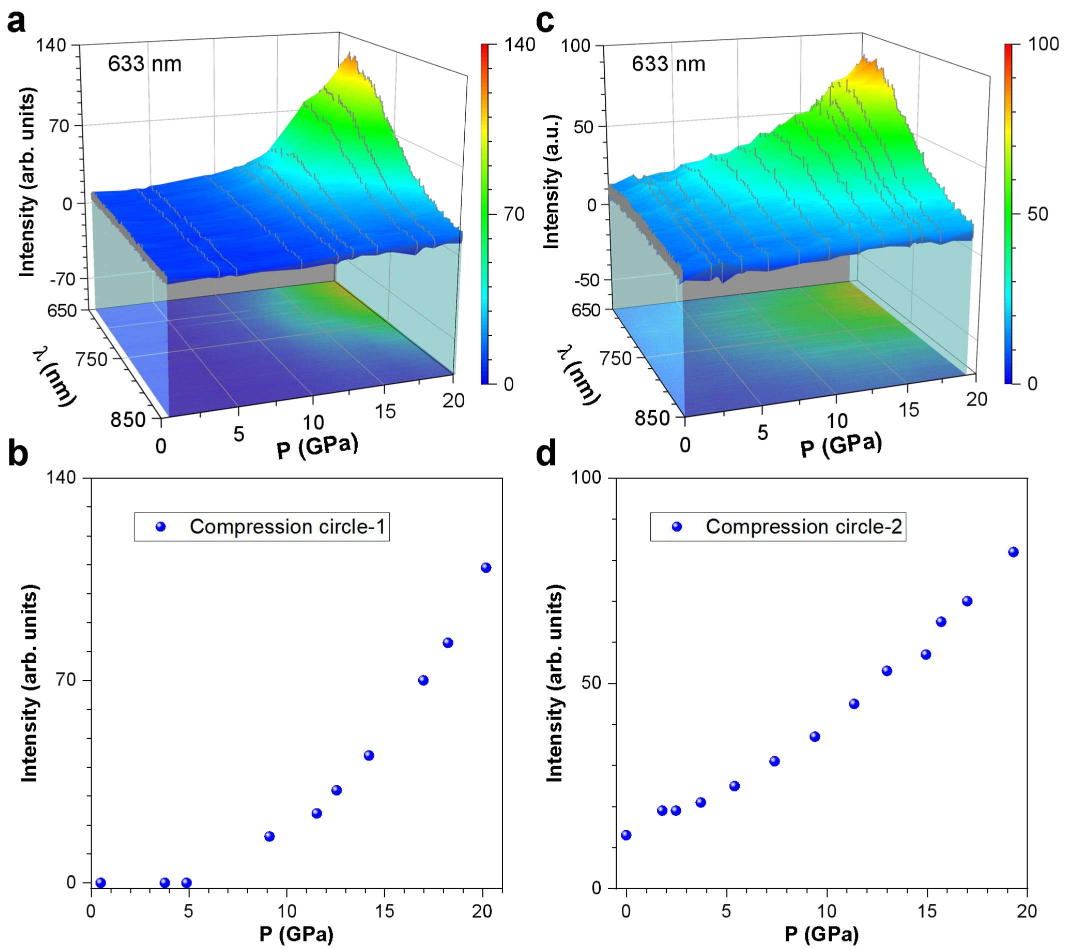

Supplementary Figure 12. The fluorescence spectra of amorphous FTPE under different pressures. 3D fluorescence spectra (**a**) and the changes in the fluorescence intensity (**b**) in the process of pressuring FTPE crystal to FTPE amorphous (from ambient pressure to 20.0 GPa) under laser excitation at 633 nm. 3D fluorescence spectra (**c**) and the changes in the fluorescence intensity (**d**) in pressuring FTPE amorphous (which is obtained after releasing from (**a**)) to 20.0 GPa under laser excitation at 633 nm.

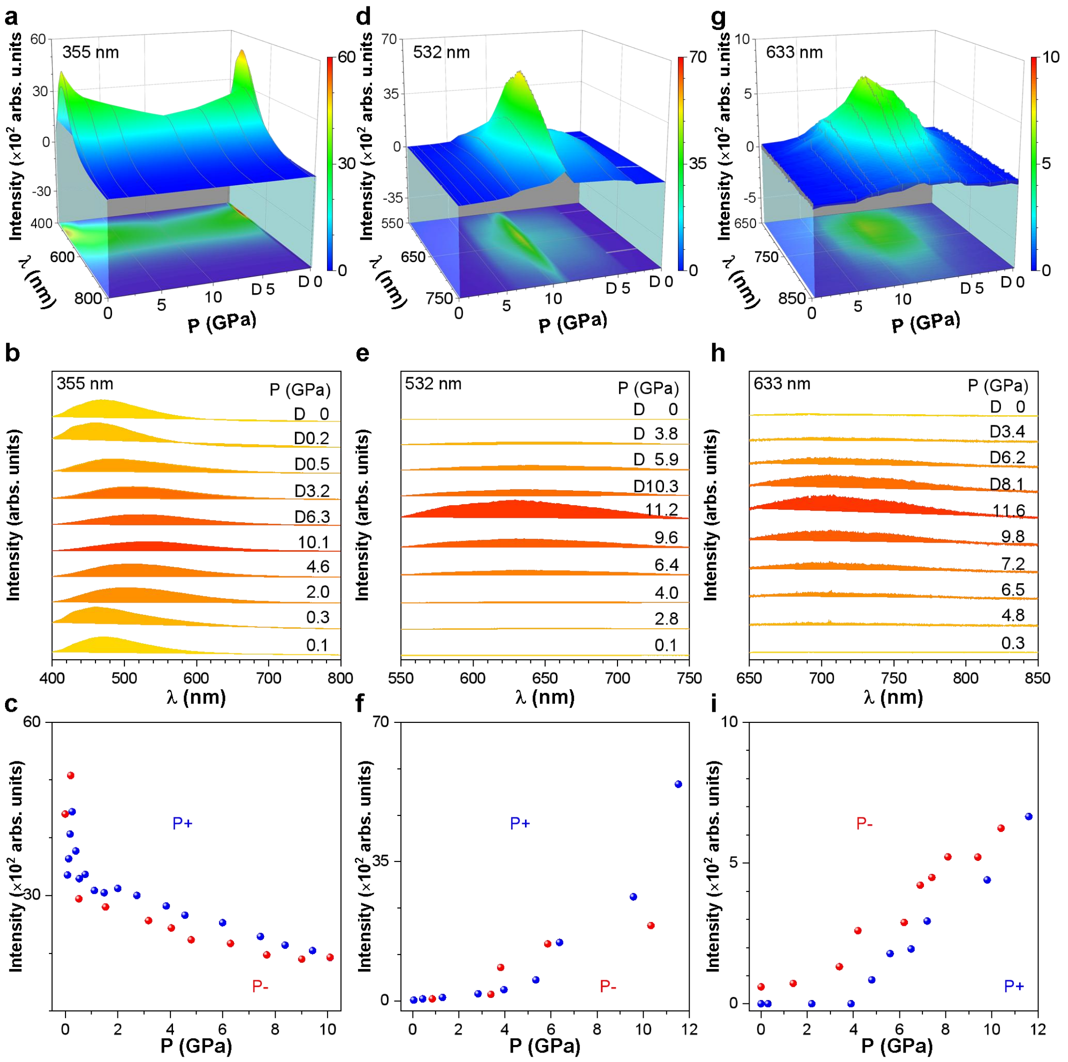

Supplementary Figure 13. Fluorescence characteristics of FTPE during the compression. Pressure-dependent 3D and 2D fluorescence spectra of FTPE single crystal during the compression (P_{max} ~ 10 GPa) and decompression under laser excitations of 355 nm (**a**, **b**), 532 nm (**d**, **e**), and 633 nm (**g**, **h**). Changes in the fluorescence intensity with pressure during the compression and decompression processes under laser excitation at 355 nm (**c**), 532 nm (**f**), and 633 nm (**i**). (D indicates the decompression process, P+ and P- in (**c**, **f**, **i**) indicate the fluorescence during the compression and decompression, respectively.)

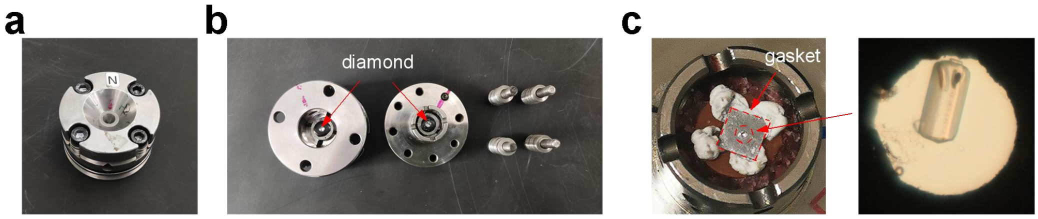

Supplementary Figure 14. The physical photos of DAC. (**a**)The assembled DAC device. (**b**) The separated DAC device. (**c**) The sample assembly drawing.

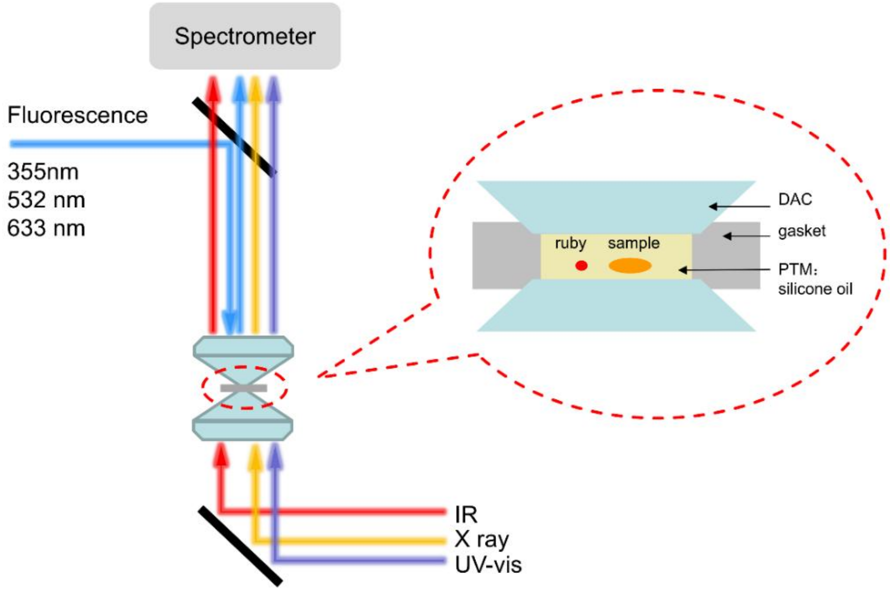

Supplementary Figure 15. Schematic diagram of the experimental setup and the sample assembly. The sample cavity was composed of diamond, gasket, ruby, pressure-transmitting medium (PTM), and sample. Ruby was inserted into the sample cavity for *in situ* pressure calibration. Oil was inserted into the sample cavity for better hydrostatic pressure.

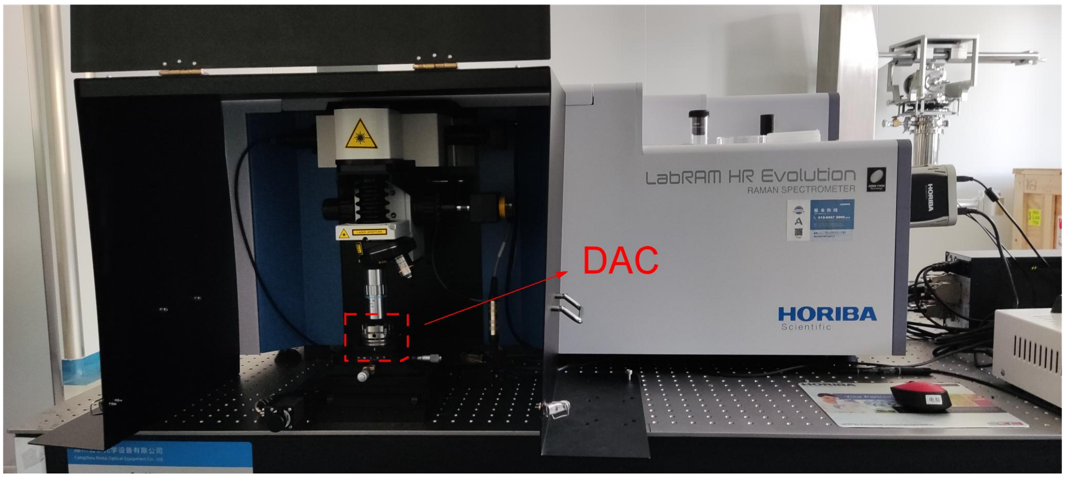

Supplementary Figure 16. **The photo of the DAC device during the fluorescence spectra collection.** The spectrometer in the phhoto is LabRAM HR Evolution, Horiba Scientific.

2. Supplementary Tables

Supplementary Table 1. Refined Structural Parameters of the FTPE under high pressure.

P (GPa)	a (Å)	b (Å)	c (Å)	β (º)	V (Å)^3	R_{wp}	χ^2
0.8	13.776	10.078	14.336	90.576	1990.150	0.0211	0.7276
1.4	13.647	9.998	14.212	91.006	1939.094	0.0210	0.7266
2.2	13.623	9.818	14.193	91.357	1897.854	0.0211	0.7293
3.1	13.428	9.672	13.972	92.850	1812.470	0.0092	0.1374
4.6	13.118	9.453	13.772	93.255	1705.115	0.0195	0.6156
5.5	13.021	9.243	13.406	92.827	1611.540	0.0089	0.1345
6.9	13.003	9.175	13.277	93.923	1580.262	0.0085	0.1634
8.0	12.877	9.109	13.133	93.474	1537.580	0.0086	0.1240
10.5	12.832	9.065	13.099	93.129	1521.478	0.0105	0.1873
11.4	12.786	8.980	13.025	92.905	1515.834	0.0086	0.1248
13.0	12.686	8.903	13.000	92.166	1467.300	0.0089	0.1348
14.5	12.650	8.900	12.920	92.200	1453.526	0.0094	0.1519

Supplementary Table 2. **IR modes of FTPE at 0.2 GPa and the calculated modes by Material Studio under atmospheric pressure.**

Calculated results/cm^{-1}	Expermental results/cm^{-1}	Assignments
722	726	γ_{C-H} (aromatic C−H out of plane bending)
730	739	γ_{C-H} (aromatic C−H out of plane bending)
765	775	γ_{C-C-C}
777	784	γ_{C-C-C} and γ_{C-H} (1,4 aromatic)
812	814	γ_{C-H} (1,4 aromatic)
818	830	γ_{C-H} (1,4 aromatic)
1014	1012	β_{C-H} (1,4/1,2 aromatic C−H in plane bending), ν_{C-O} (CH_3−O stretching), δ_{C-H} (CH_3 deformation vibration)
1017	1029	β_{C-H} (1,2 aromatic)
1021	1032	β_{C-H} (1,4 aromatic), ν_{C-O} (CH_3−O), δ_{C-H} (CH_3)
1071	1087	β_{C-H} (1,2 /1,4 aromatic)
1093	1107	β_{C-H} (1,4 aromatic)
1115	1135	β_{C-H} (1,2 aromatic)
1132	1153	β_{C-H} (1,2 aromatic), r_{C-H} (CH_3 rocking vibration)
1135	1154	r_{C-H} (CH_3)
1153	1171	β_{C-H} (1,4 aromatic), r_{C-H} (CH_3)
1161	1177	β_{C-H} (1,4 aromatic), r_{C-H} (CH_3)
1168	1185	r_{C-H} (CH_3)
1181	1189	β_{C-H} (1,2 aromatic), r_{C-C}
1239	1246	β_{C-H} (1,4 aromatic), ν_{C-O} (aromatic C−O), r_{C-H} (CH_3)
1249	1252	β_{C-H} (1,4 aromatic), ν_{C-O} (aromatic C−O), r_{C-H} (CH_3)
1264	1277	β_{C-H} (1,4 and 1,2 aromatic), γ_{C-C-}
1293	1291	β_{C-H} (1,4 and 1,2 aromatic)
1315	1319	β_{C-H} (1,4 aromatic), γ_{C-C-C}, r_{C-H} (CH_3)
1424	1435	δ_{C-H} (CH_3), β_{C-H} (1,2 aromatic)
1428	1446	β_{C-H} (1,2 aromatic), δ_{C-H} (CH_3)
1438/1441	1458	r_{C-H} (CH_3)
1453/1455	1474	β_{C-H} (1,2 aromatic), δ_{C-H} (CH_3)
1486/1490	1506	β_{C-H} (1,4 aromatic), δ_{C-H} (CH_3)
1543/1548	1559	β_{C-H} (1,4 aromatic), γ_{C-C-C}
1559	1572	$\gamma_{C=C}$

1585	1602	β_{C-H} (1,4 aromatic), γ_{C-C-C}
1592	1616	β_{C-H} (1,2 aromatic), γ_{C-C-C}, $\gamma_{C=C}$
2926	2837	ν_{C-H} (CH_3)
2938	2844	ν_{C-H} (CH_3)
3007	2931	ν_{C-H} (CH_3)
3032	2945	ν_{C-H} (CH_3)
3053	2973	ν_{C-H} (CH_3)
3069	3001	ν_{C-H} (1,2 aromatic)
3090	3030	ν_{C-H} (1,2 aromatic)
3103	3055	ν_{C-H} (1,2 aromatic)
3121	3070	ν_{C-H} (1,4 aromatic)
3136	3093	ν_{C-H} (1,4 aromatic)
